# Encapsulation of Vitamin E in Yogurt-Based Beverage Emulsions: Influence of Bulk Pasteurization and Chilled Storage on Physicochemical Stability and Starter Culture Viability

**DOI:** 10.3390/molecules26061504

**Published:** 2021-03-10

**Authors:** Vassilios Raikos, Lynn P. Pirie, Sati Gürel, Helen E. Hayes

**Affiliations:** 1 Rowett Institute, University of Aberdeen, Aberdeen, Scotland AB25 2ZD, UK; l.p.pirie@abdn.ac.uk (L.P.P.); satigurel@trakya.edu.tr (S.G.); h.hayes@abdn.ac.uk (H.E.H.); 2 Department of Nutrition and Dietetics, Health Science Faculty of Trakya University, 22030 Edirne, Turkey

**Keywords:** yogurt, vitamin E, emulsion, encapsulation, antioxidant activity

## Abstract

Yogurt is a nutritious food that is regularly consumed in many countries around the world and is widely appreciated for its organoleptic properties. Despite its contribution to human dietary requirements, yogurt in its traditional recipe is a poor source of fat-soluble vitamins. To respond to consumer demands and further increase the nutritional value of this product, this work aimed to fortify yogurt with vitamin E by using emulsification as the method of encapsulation. The effects of thermal processing and chilled storage on the physicochemical stability of the yogurt-based beverage was investigated. Vitamin E was only minorly affected by bulk pasteurization at 63 °C for 30 min and remained stable during storage at 4 °C for 28 days. Fortified samples showed increased in vitro antioxidant activity compared with non-fortified samples. Lactic acid bacterial counts were above the minimum recommended levels (>10^6^ cfu/g) after processing and storage. In conclusion, this work has demonstrated that emulsification can be an effective strategy for developing yogurt-based products fortified with fat soluble vitamins.

## 1. Introduction

Yogurt is a traditional food staple produced by the fermentation of milk with the lactic acid bacteria *Streptococcus thermophilus* and *Lactobacillus delbrueckii* spp. bulgaricus [1]. Yogurt is a valuable source of energy, macro- and micronutrients, primarily protein, calcium and B vitamins, and thus is considered an important element of the human diet in many countries around the world [2]. In addition, the moderate consumption of fermented foods (i.e., yogurt) is believed to offer additional health benefits beyond nutrition, which are largely attributed to the delivery of probiotic bacteria to the human gastrointestinal tract [3].

The exact nutritional composition of commercially available yogurt depends on numerous variables such as formulation, processing and storage and is therefore product-specific [4]. Despite its desirable nutritional value, yogurt is generally considered to be a relatively poor source of fat-soluble vitamins, particularly D and E, which suggests that its consumption makes a minor contribution to the daily recommended intake of the above-mentioned micronutrients. Vitamin D is a vitamin with hormone function and its role in the homeostasis of calcium, phosphorus and bone metabolism is well known, whereas the term vitamin E refers to a group of fat-soluble compounds formerly known as tocopherols and tocotrienols with a well-documented antioxidant activity [5,6].

To overcome this deficit and further increase the nutritional value of the end product, food manufacturers often add a vitamin fortification step in the process of yogurt manufacture, typically including vitamin D [7]. Although food fortification with fat soluble vitamins is an appealing strategy for developing products with enhanced nutritional and health benefits, the process comes with its own challenges. This is because fat soluble vitamins are not be compatible with aqueous-based food matrices and/or are easily degraded by environmental factors (i.e., light, oxygen, heating, etc.), which limits their absorption and bioavailability [8]. Considerable efforts have been made to identify suitable encapsulation methods for optimum delivery of vitamin D using yogurt as food matrix [9,10]. However, supplementation of yogurt with vitamin E is scarce and is often facilitated using alternative product development approaches [11].

Emulsification is considered an advantageous encapsulation approach to ensure fat soluble vitamins maintain their physical and chemical integrity during the production stages and therefore can be delivered at nutritionally beneficial doses [12]. Previous work has indicated that emulsion-based food systems can be used effectively as “vehicles” for vitamin E encapsulation, offering protection against processing and storage effects [13,14,15]. A fermented beverage emulsion system using goat milk proteins as emulsifiers and lactic acid bacteria as starter culture, was recently developed and characterized [16]. In this study, a similar formulation approach was adopted to fortify a yogurt-based beverage with vitamin E using emulsification as the encapsulation method. The purpose of this study was to investigate the effect of bulk pasteurization and chilled storage on the physicochemical and microbiological stability of the vitamin E-fortified yogurt-based beverage. To the best of our knowledge, this is the first study to report the processing effects on the properties of a fermented dairy-based emulsion fortified with vitamin E.

## 2. Results and Discussion

### 2.1. Processing and Storage Effects on Tocopherol Content and Antioxidant Capacity

This work describes the development of a yogurt-based beverage fortified with vitamin E and examines the effects of thermal processing and chilled storage on selected quality attributes. The recipe was tailored to ensure that the daily recommended intake of vitamin E for adults (15 mg) [17] is provided when consuming 60 g of the yogurt drink. Table 1 presents the effect of pasteurization and cold storage on tocopherol content of the yogurt-based beverages. Data indicate that mild thermal processing (bulk pasteurization) has a minimal effect on tocopherol content. Pasteurized yogurt beverages contain approximately 7% less vitamin E than unpasteurized ones. This suggests that, although vitamin E is susceptible to heating-induced degradation [18], the selected processing conditions do not have a significant effect (*p* > 0.05). This finding is consistent with published data, which denote that retention of vitamin E in emulsions is high (>77%) when processing occurs at mild heat treatments [15] and degradation rate significantly increases at temperatures above 75 °C [19]. Similarly, cold storage at 4 °C for 28 days resulted in moderate, non-significant losses of vitamin E. Pasteurized samples showed a decrease of 7.7% and unpasteurized ones of 11.6% (*p* > 0.05). Previous work has shown similar degradation pattern of vitamin E in cheese samples [20] and frozen yoghurt [21] under refrigerated and frozen storage respectively and results indicated that storage under light is less favorable [20]. Vitamin E degradation during cold storage is attributed to oxidation induced by the presence of oxygen in yogurt and this effect can be accelerated by light, heat, alkali, trace minerals and hydroperoxides [22].

**Figure 1:**
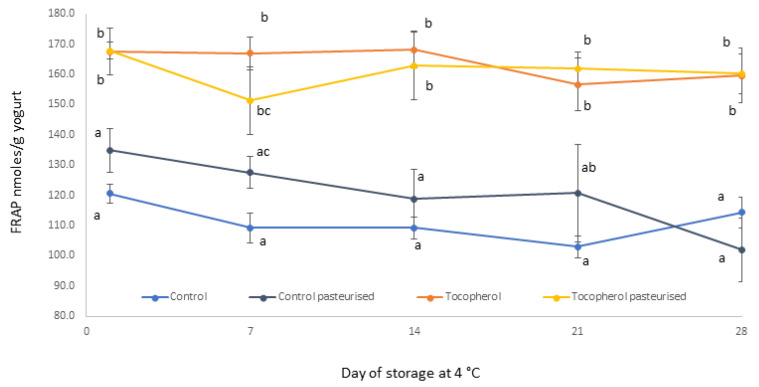
FRAP (ferric reducing antioxidant power) of yogurt-based beverages during cold storage. Results are presented as mean ± SD. Different low case letters denote significant differences at each time point (*p* < 0.05).

FRAP (ferric reducing antioxidant power) assay is commonly used to assess antioxidant activity of food samples in vitro. The assay determines the ferric reducing activity of food extracts under controlled conditions and α-tocopherol is often used as a reference compound with documented antioxidant activity [23]. The radical scavenging activity of tocochromanols (compounds with vitamin E activity) is attributed to the phenolic hydrogen of the chromanol ring, which is donated to lipophilic free radicals [24]. Figure 1 shows data from the antioxidant capacity of the yogurt-based beverages during chilled storage as determined by FRAP. Samples fortified with vitamin E, both pasteurized and unpasteurized, show significantly higher (*p* < 0.05) antioxidant activity compared with non-fortified samples at time points 1, 14 and 28 days. This effect is consistent throughout the storage period and is largely attributed to vitamin E content of fortified samples. As depicted in Figure 1, non-fortified samples show considerable antioxidant properties. Previous studies have demonstrated that fermented milk products contain peptides with antioxidant activity [25]. These are released during the fermentation process and typically contain phenolic side chains (e.g., tyrosine) [26]. In addition, phenolic compounds, products of the secondary metabolism of plants, are naturally present in milk and derive from ruminant feed [27]. The increase in total tocopherol content (α- β- and δ-tocopherol) shows a strong correlation with the increase in antioxidant activity between fortified and non-fortified samples during storage (R^2^ = 0.87). This correlation suggests that fortification with vitamin E markedly increases the antioxidant activity of the fermented beverage emulsion and this effect is largely unaffected by pasteurization and chilled storage. Published studies have demonstrated significant correlation between vitamin E content of wheat, vegetable oils, milk and milk cream and their measured antioxidant capacity [28]. The effect of storage time for each treatment is non-significant, which suggests that fortified samples retain their antioxidant capacity for at least 28 days. Furthermore, pasteurization treatment showed no significant effect on the antioxidant activity as indicated by the data. Previous research has indicated that pasteurization can affect the antioxidant properties of yogurt; however, this was attributed to the impact of thermal processing on the interactions of plant phenolics with milk proteins [29].

### 2.2. Processing and Storage Effects on Viability of Lactic Acid Bacteria

**Figure 2:**
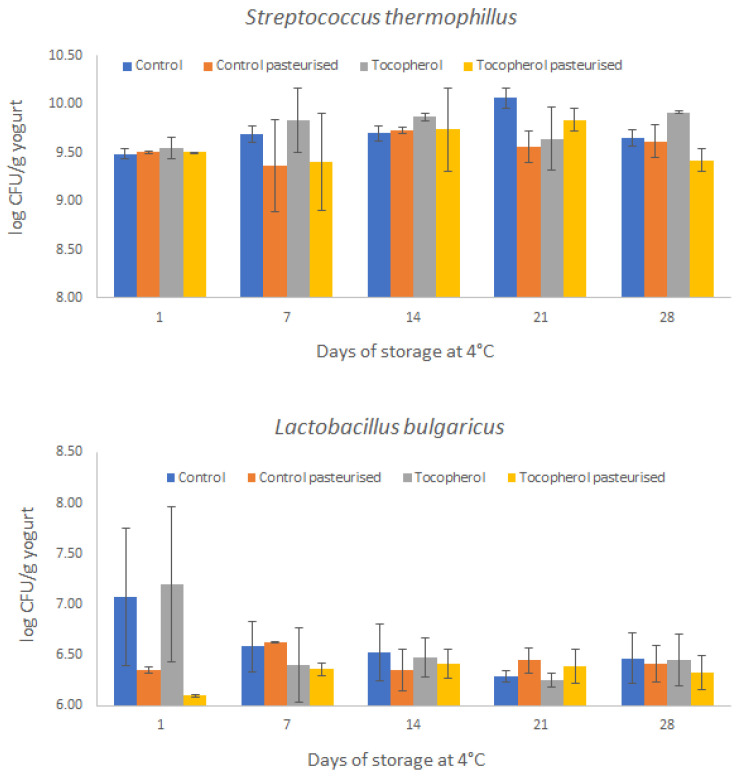
Viable counts of *S. thermophilus* and *L. bulgaricus* in yogurt-based beverages during 28 days of storage at 4 °C. Each point is the mean of two replicates from each batch (n = 4).

**Figure 3:**
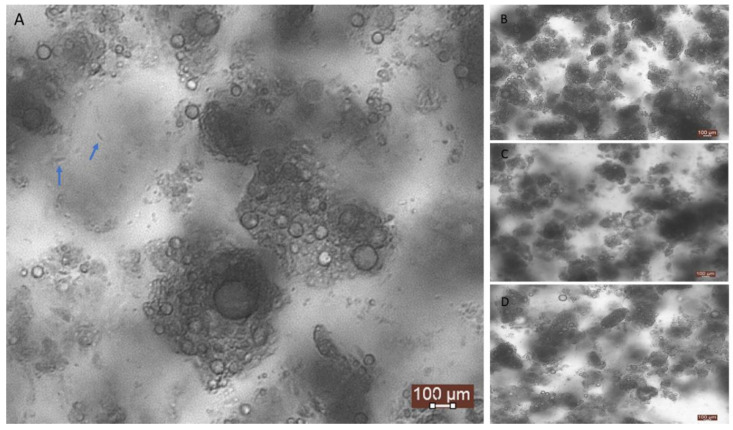
Optical microscopy images of yogurt-based beverages after 28 days of storage. (**A**) Control, (**B**) Control pasteurised, (**C**) Tocopherol and (**D**) Tocopherol-pasteurised. Arrows indicate the presence of lactic acid bacteria. Scale bar represents 100 μm.

Viability of lactic acid bacteria during the storage period of yogurt is considered an essential key attribute of product quality. Although maintaining a minimum therapeutic dose of 10^6^ cfu/g until the end of product shelf-life is desirable for exerting a probiotic effect on human health [30], this target is not always achievable [31]. Most commonly used probiotic strains used in yogurt manufacture show sensitivity to low pH, which affects their long-term survival in yogurt [32]. The shelf-life of most yogurt products varies between 20–40 days under refrigerated conditions and processing conditions, particularly severe heat treatment, can have detrimental effects on lactic acid bacteria viability [33]. Dairy products are commonly subjected to pasteurization, which aims to prolong shelf-life and prevent post-acidification processes [34]. Hence, a pasteurization step was added in the manufacturing process of the yogurt-based beverage emulsions. The viability of lactic acid bacteria (*Streptococcus thermophilus* and *Lactobacillus delbrueckii* spp. Bulgaricus) during 28 days of chilled storage is presented in Figure 2. Data indicates that viability of both lactic acid bacteria species is maintained above desirable levels (>10^6^ cfu/g) after the storage period. Visual observations at the end of the storage period also confirmed that lactic acid bacteria remained abundant and viable, as shown in Figure 3. The counts of *Streptococcus thermophilus* (~9.5 log cfu/g) were higher than those of *Lactobacillus delbrueckii* spp. Bulgaricus (~6.7 log cfu/g) throughout the storage period. Thermal processing affected only *Lactobacillus delbrueckii* spp. Bulgaricus as indicated by a reduction of 12.8% in viable counts at day 1; however, this effect was non-significant (*p* > 0.05). According to Luana et al. [35] pasteurization at 63 °C for 30 min decreased lactic acid bacteria counts from 6.5 × 10^8^ cfu/mL to 2.0 × 10^6^ cfu/mL (approximately 2 log cycles) in a yogurt-like beverage and thus the starter survived at a relatively high number [35]. Previous research has indicated that non-standard heat treatment at 63 °C for 5 min did not affect the number (>8.0 log cfu/g) of both bacterial species after 21 days of storage [36]. Present findings suggest that both bacterial species can withstand heat treatment at 63 °C for 30 min without significant losses in viability under refrigerated storage for 28 days.

### 2.3. Processing and Storage Effects on Physical Stability

**Figure 4:**
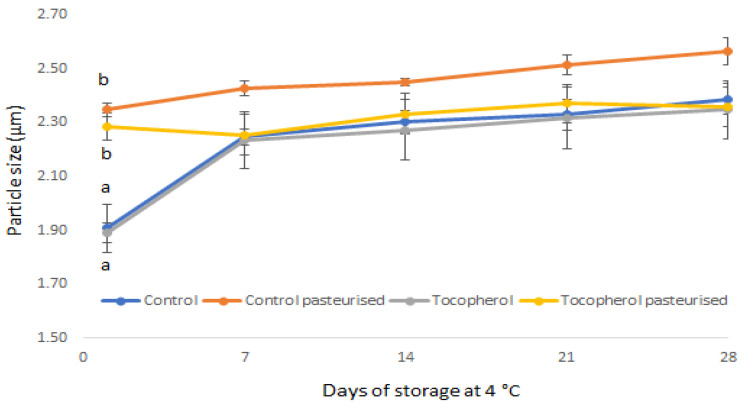
Particle size of oil droplets in yogurt-based beverages during refrigerated storage (28 days). Values are the mean ± SD.

Homogenization and heat treatment are common processing steps in conventional yogurt manufacturing process, which precede fermentation and affect microstructure [37]. Homogenization facilitates the breakdown of lipid droplets resulting in increased surface area and facilitates casein and whey protein adsorption at the interface with subsequent formation of a surface layer around the fat globules [38]. Heat treatment induces structural changes to milk proteins (i.e., whey protein denaturation) and facilitates inter-molecular interactions between caseins and whey proteins [39]. The formation of the typical three-dimensional network consisting of clusters, chains and strands of associated casein particles is mediated by acid gelation during fermentation [40]. The microstructure of the yogurt-based beverages is shown in Figure 3. Optical microscopy analysis revealed the formation of a water-in-oil emulsion system, made up of small clusters of associated oil droplets and void spaces in which the aqueous phase is dispersed. Microscopic data was unable to detect any structural changes between heat-treated and untreated samples. Multiple light scattering (Turbiscan analysis), which enables continuous monitoring of the optical properties of undiluted emulsion systems and provides real-time information on destabilization phenomena, was employed to determine the processing and storage effects on physical stability [41]. Particle size analysis showed that thermal processing had a significant incremental effect on oil droplet size at day 1 (Figure 4). This effect may be attributed to whey protein denaturation (adsorbed and non-adsorbed), with subsequent thiol group exposure leading to interactions with other denatured whey molecules in proximity and/or caseins (mostly κ- and α_s1_-) [42]. These disulfide-mediated interactions can account for the incremental effect on particle size due to droplet flocculation [43]. Previous studies have demonstrated that depletion flocculation, induced by heat treatment, is the main destabilization mechanism of sodium caseinate stabilized emulsions [44]. However, this effect is not significant after 7 days at 4 °C and until the end of storage period. It seems that heat treatment may accelerate the process of droplet aggregation, which would occur upon cooling and chilling. However, there was no visual separation of the oil phase at the end of storage for all samples, which signifies that homogenization was effective in preventing any creaming effects. Creaming defects may also be limited due to the formation of a jellified network which reduces fat mobility (Figure 3) and can also be attributed to the low-fat content (5%) [45]. Improved creaming stability in heated emulsions may also be due to the combined effect of increased surface protein coverage, higher continuous phase viscosity and the presence of aggregates in the continuous phase [46].

## 3. Materials and Methods

### 3.1. Materials/Microorganisms

Spray-dried goats milk powder (skimmed) and yogurt culture (Yo-Mix^®^ ABY) were purchased from Goat Nutrition Ltd. (Ashford, England). Corn oil was purchased from the local supermarket (Tesco, UK). Tocopherols mixed were purchased from Sigma Aldrich (St. Louis, MO, USA). All reagents used were of analytical grade.

### 3.2. Preparation, Processing and Storage of Yogurt-Type Beverage

Yogurt-based beverages were prepared using the following weight recipe (78.75% water, 16% goat milk powder, 5% corn oil, 0.25% freeze dried culture). For the vitamin E-fortified samples the recipe was slightly modified by adjusting the water content. Mixed tocopherols (0.06%) were added to corn oil and the mixture was agitated until fully dissolved. Goat milk powder was initially reconstituted in water to allow hydration for 20 min with little agitation. A coarse emulsion (500 g) was formed by adding corn oil (and mixed tocopherols if applicable) at a steady rate and mixing the remaining of the ingredients with a high-speed hand blender (Morphy Richards, Argos, UK) for 2 min. Homogenized samples were pasteurized using a Klarstein Biggie Digital fully automatic cooker at 63 °C for 30 min. 1.25 g (~9 Direct Culture Unit) of the freeze-dried mixed culture were added to the beverage and mixed thoroughly. Samples were then poured in a sterile glass container and were placed in a 7-Cup Electric Yogurt Maker (Lakeland, Aberdeen, UK) set at 43 °C. The fermentation process was monitored every hr using a portable food and dairy pH meter (Hanna Instruments Ltd., Leighton Buzzard, UK) and was terminated when pH reached 4.5 (~6 h). Yogurts were then diluted (1:1) with water, mixed with the hand blender for 2 min and were further homogenized by passing the samples through a high-pressure homogenizer (APV-1000, SPX Flow Technology, West Sussex, UK) at 100 MPa for 5 times. All samples were then stored at 4 °C for 5 weeks before further analysis (sampling was performed on a weekly basis). Two batches of fermented yogurt-based beverages were prepared and used for subsequent analysis.

### 3.3. Tocopherol Analysis

Alpha-, gamma- and delta-tocopherols concentrations in yogurt-based beverages were determined by reverse phase HPLC, using fluorescence and visible detections according to the method described by Hess et al. [47]. HPLC analysis of the extracts was performed using a Waters 717 plus Autosampler Module (Waters Corporation, Milford, USA) equipped with a Waters 2475 scanning fluorescence detector, a 2487 UV/VIS absorbance detector and a C-18 silica (Beckman Ultrasphere ODS) analytical column (250 × 4.6 mm ID 5 mm particle size) using the following settings: elution flow rate: 1.2 mL/min, sample run: 30 min and injection volume: 150 mL. Quantification was carried out against mixed standards (Sigma Aldrich) containing all tocopherols at appropriate concentrations and results were expressed in μg/mL of yogurt beverage. Echinone was used as an internal standard.

### 3.4. Particle Size Determination

The physical stability of yogurt-based beverages was monitored using a Turbiscan MA2000 (Formulaction, Ramonville St. Agne, France). The apparatus comprises of a detection head equipped with a near-infrared light source (880 nm) which scans the length of the sample, acquiring transmission and backscattering data every 40 mm. The light source scanned the sample at 5 min intervals from top to bottom and measured the percentage of light backscattered or transmitted during 1 h period at 25 °C. Backscattering profiles (%ΔBS) from the middle of the tube (10–30 mm) were used to monitor size variation coalescence/flocculation) phenomena for particle size determination by the Turbisoft Lab 2.2 software. The refractive indices of the dispersed and continuous phase which were used to compute the mean spherical equivalent diameter were 1.47 and 1.33 respectively.

### 3.5. Microbiological Analysis

Sampling for lactic acid bacteria was performed during the chilled storage at weeks 1-5. *Lactobacillus bulgaricus* was inoculated in Lactobacillus selective agar (83920 Rogosa Agar, Sigma-Aldrich, Dorset, UK) and incubated at 37 °C for 4 days and *Streptococcus thermophilus* was cultivated in M-17 agar (63016, Sigma-Aldrich, Dorset, UK) and incubated at 37 °C for 2 days. The total viable microbial populations capable of growing on agar plates (20–200 CFUs) were analyzed in duplicate and the average values of colony forming units (log CFU/g) are presented.

### 3.6. Optical Microscopy

Light micrographs of the yogurt-based beverages were obtained using a Leica DM IL LED inverted laboratory microscope equipped with a Leica DFC295 digital color camera (Leica microsystems Ltd., Milton Keynes, UK). The samples were observed through a 40×dry objective lens. Pictures were taken using the in-built 3 MP digital camera and picture analysis was performed using Leica application suite software (V.3.6.0).

### 3.7. Antioxidant Activity (FRAP)

Yogurt samples (1.0 g) were extracted with 19 mL ethanol (50%) for 2 h with shaking at room temperature. Samples were then centrifuged at 3220× *g* (Eppendorf^®^5810R, Fisher Scientific, Loughborough, UK) for 1 h at 4 °C and the supernatant was collected. The extraction was repeated twice, the supernatants were combined and dried using a rotary evaporator (Rotavapor R-114, Büchi Labortechnik AG, Switzerland) and the remaining solution was freeze-dried (FreeZone, Labconco Corporation, MO, USA). The dried extract was reconstituted in DMSO (100%) at 200 mg/mL, aliquoted and stored at −20 °C until further analysis. The total antioxidant potential of the yogurt beverage samples was determined using the FRAP assay adapted from Benzie and Strain [48] as a measure of antioxidant power. The FRAP reagent was freshly prepared by mixing 25 mL of acetate buffer (300 mM, pH 3.6), 2.5 mL of a solution of 10 mM TPTZ in 40 mM HCl and 2.5 mL of 20 mM FeCl_3_ and once prepared was kept in a water bath at 37 °C. Sample extract and standard were diluted 1:4 with water and the following amounts were added directly in a 96 well plate and absorbance was measured using a SpectraMax 190 microplate reader (Molecular Devices Limited, Berkshire, UK) thermostated at 37 °C: 180 μL FRAP reagent and 24 μL of the extract, standard or H_2_O as a blank. Absorbance readings were taken at 593 nm after 10 min at 37 °C. Serial dilutions of a 1 mM FeSO_4_ solution were used to prepare the standard curve. The results were corrected for dilution and expressed in nmoles Fe(II) equivalents per g of yogurt.

### 3.8. Statistical Analysis

All experiments were conducted on at least one replicate from each batch (*n* ≥ 3). Results are expressed as means ± standard deviation (SD) of at least three replicates. Data were subjected to statistical analysis by SPSS version 25 software. The normality of data distribution was tested by the Shapiro–Wilk method. Statistical significance values of groups’ means were made by repeated measures analysis of variance (rmANOVA) for repeated measurements (weeks 1–5). The Bonferroni post hoc test was used to detect statistically significant results. The statistical analysis performed was considered significant when *p* < 0.05.

## 4. Conclusions

This research is a proof of concept study for the development and characterization of a yogurt-based beverage emulsion fortified with vitamin E. Results signify that vitamin E is well-retained in the dispersed phase of the oil-in-water emulsion post-pasteurization and after chilled storage for 28 days. Vitamin E-fortified beverage emulsions showed significantly increased antioxidant capacity in vitro compared with the non-fortified counterparts and this effect was consistent during storage. Lactic acid bacteria viability was largely unaffected by thermal processing and storage for all formulations and their abundance was above the minimum recommended levels for such products after 28 days at 4 °C. Droplet size showed a moderate incremental trend with heat treatment and storage time which should have a minor effect on product stability for the specified shelf-life. Overall, this study confirms that emulsification can be used as an effective strategy to encapsulate vitamin E in yogurt formulations. Mild heat treatment and chilled storage conditions do not have any significant, negative effects on product’s physicochemical and microbiological stability. The findings of this study can be further exploited by the food and drink industry for the development of fermented foods with desirable nutritional profiles.

## Figures and Tables

**Table 1:** Effect of pasteurization on tocopherol content (μg/mL) of the yogurt-based beverages during cold storage over a period of 28 days.

Days of Storage at 4 °C		1	7	14	21	28
α-tocopherol	Control	ND	ND	ND	ND	ND
	Control-P	ND	ND	ND	ND	ND
	Tocopherol	16.98 ± 2.9 ^a^	18.37 ± 0.6 ^a^	12.40 ± 1.7 ^a^	13.27 ± 2.9 ^a^	13.35 ± 3.5 ^a^
	Tocopherol-P	14.99 ± 1.3 ^a^	16.95 ± 3.0 ^a^	13.13 ± 1.0 ^a^	14.01 ± 1.1 ^a^	12.87 ± 1.3 ^a^
γ-tocopherol	Control	0.17 ± 0.0 ^a^	0.21 ± 0.0 ^a^	0.15 ± 0.0 ^a^	0.16 ± 0.0 ^a^	0.17 ± 0.0 ^a^
	Control-P	0.15 ± 0.0 ^a^	0.12 ± 0.0 ^a^	0.10 ± 0.0 ^a^	0.07 ± 0.0 ^a^	0.12 ± 0.0 ^a^
	Tocopherol	169.52 ± 27.0 ^b^	177.15 ± 7.4 ^b^	132.95 ± 19.5 ^b^	143.90 ± 29.4 ^b^	148.33 ± 33.8 ^b^
	Tocopherol-P	156.75 ± 12.4 ^b^	165.78 ± 32.5 ^b^	139.43 ± 14.6 ^b^	146.77 ± 13.7 ^b^	144.54 ± 15.2 ^b^
δ-tocopherol	Control	0.02 ± 0.0 ^a^	0.02 ± 0.0 ^a^	0.02 ± 0.0 ^a^	0.01 ± 0.0 ^a^	0.02 ± 0.0 ^a^
	Control-P	0.02 ± 0.0 ^a^	0.02 ± 0.0 ^a^	0.01 ± 0.0 ^a^	0.01 ± 0.0 ^a^	ND
	Tocopherol	39.79 ± 4.7 ^b^	41.99 ± 2.0 ^b^	33.86 ± 4.4 ^b^	35.98 ± 7.0 ^b^	36.25 ± 8.0 ^b^
	Tocopherol-P	38.81 ± 2.7 ^b^	39.40 ± 7.6 ^b^	35.05 ± 3.5 ^b^	36.86 ± 3.2 ^b^	36.92 ± 3.5 ^b^

Values with different subscript are significantly different (*p* < 0.05); ND: not determined due to the detection limit of the method.

## Data Availability

Not applicable.
